# Research funders should be more transparent: a plea for open applications

**DOI:** 10.1098/rsos.220750

**Published:** 2022-10-12

**Authors:** Serge P. J. M. Horbach, Joeri K. Tijdink, Lex Bouter

**Affiliations:** ^1^ Danish Centre for Studies in Research and Research Policy, Aarhus University, Bartholins Allé 7, 8000 Aarhus, Denmark; ^2^ Department of Ethics, Law and Humanities, Amsterdam University Medical Centers, Vrije Universiteit, De Boelelaan 1105, 1081 HV Amsterdam, The Netherlands; ^3^ Department of Epidemiology and Data Science, Amsterdam University Medical Centers, Vrije Universiteit, De Boelelaan 1105, 1081 HV Amsterdam, The Netherlands; ^4^ Faculty of Humanities, Department of Philosophy, Vrije Universiteit, De Boelelaan 1105, 1081 HV Amsterdam, The Netherlands

**Keywords:** research funding, responsible research practices, Open Science, transparency

## Abstract

Transparency is increasingly becoming the new norm and modus operandi of the global research enterprise. In this mini-review, we summarize ongoing initiatives to increase transparency in science and funding in particular. Based on this, we make a plea for the next step in funders' compliance with the principles of Open Science, suggesting the adoption of *open applications*. Our proposed model includes a plea for the publication of all submitted grant applications; open sharing of review reports, argumentations for funding decisions and project evaluation reports; and the disclosure of reviewers' and decision committee members' identities. In line with previous calls for transparency and the available evidence about these measures' effectiveness, we argue that open applications could lead to more diverse collaboration, recognition of research ideas, fairer procedures for grant allocation, more research on funding practices and increased trust in the funding allocation process.

## 1. Research funders should be more transparent: a plea for open applications

Open Science is increasingly envisioned to be the future of the global research enterprise [1–3]. Consequently, multiple aspects of research have progressed towards more transparency. Research funders are increasingly joining the movement towards more openness by mandating the researchers and organizations they fund to engage in open data, open access publication or public registration of full study protocols prior to a study's start [4,5].

In this mini-review, we make a plea for the next step in funders' compliance with the principles of Open Science, suggesting the adoption of *open applications*. Subsequently, we embed this proposal in the academic literature by summarizing ongoing discussions and efforts with the aim to increase transparency in academic funding. Our proposed next step entails transparency about all grant applications—publishing both funded and unfunded applications—the assessment of their relevance and quality, and the decision and monitoring process. In this review, we take stock of earlier initiatives and discuss the arguments in favour of and against their implementation and extension.

## 2. Our proposal

A more open system of grant application should address multiple aspects. Firstly, it should encompass open sharing of applications—both funded and unfunded ones—either directly after the submission deadline, after grant decisions were made or at some later stage. Secondly, it should include open sharing of review reports and of the argumentation that led to the funding decision, potentially later followed by monitoring and evaluation reports of funded projects. Thirdly, we suggest the open sharing of reviewers' and decision committee members' identities.

While the evidence on the effects of the last aspect, the sharing of identities, is still inconclusive [6,7], we argue that the first two features present clear benefits to the research system and can be implemented relatively quickly. The first suggestion, the sharing of all applications, has several prominent advantages. It will provide additional opportunities for collaboration and the usage of research ideas by others, including in other contexts than initially envisioned [2]. It enables cross-fertilization of research ideas and has the potential to engage additionally relevant, but perhaps overlooked, project partners or disciplines. In addition, we reiterate that increasingly many organizations require the work of publicly funded researchers to be freely available to the public. We argue that grant applications constitute an important part of this.

The second suggestion, open sharing of review reports, decision argumentations, and later monitoring and evaluation reports, will provide opportunities for learning and can ultimately support the development and improvement of future proposals by the same applicant or others. The level of transparency will ideally not only make funding decisions more understandable and more acceptable, but also enable a check whether the procedures specified in the call for applications were followed and the review process was fair. This improved level of transparency will likely increase trustworthiness of the way proposals are handled and of the grant allocation system in general. In addition, a system of open applications will stimulate and enable research on research funding practices, allowing better understanding of, and ultimately improving, this essential step in the process of knowledge production. In order to facilitate sharing and improve the findability of published proposals and related documents, we advocate funders sharing them on public repositories, rather than their own webpages. Several currently existing repositories already have the infrastructure in place to implement this.

Obviously, the open sharing of grant applications can also be achieved independent of funders' support as applicants themselves can provide access to their proposals. Some initiatives to facilitate this have recently emerged, including the Open Grants platform [8]. Nevertheless, we propose research funders to become actively involved and to lead the change towards transparency. In a more radical adoption of our proposal, this could even evolve to a system in which grant applications are not submitted to a single research funder, but instead are posted to a central repository from which funders subsequently select applications they want to support. This resembles the dynamics currently emerging on some preprint servers [9], where editors of scholarly journals invite preprint authors to submit their work for peer review. A similar system is currently used for crowd-sourced research funding, which inherently relies on grant applications being openly available [10]. However, crowd-sourced research funding so far tends to be restricted to relatively small projects requiring limited budgets.

## 3. Potential objections

Of course, some obvious objections can be, and have been [11], raised against (too drastic) openness and transparency in research funding processes. Discussions tend to revolve around two main sets of objections. The first set includes arguments regarding the fear of being ‘scooped’ when applications, including unsuccessful ones, are made publicly available upon submission or at some later stage, i.e. the fear of other researchers taking off with your research ideas before you have had the time or opportunity to execute them yourself.

However, we would actually argue the opposite. If applications are published even if they are not funded, this provides a time stamp to the work, settling potential priority conflicts and providing ‘proof' of being first. In addition, having non-funded proposals out in the open increases opportunities for collaboration and usage of project ideas by others or in other contexts—with or without the initiator being involved—thereby adding value to the work put in the grant application. The Dutch Research Council (NWO) argues similarly in their rationale for publishing grant applications in the Open Science Fund [12]. If non-funded applications are published, it also gives other public or private funders the opportunity to scrutinize these research ideas and see whether they fit the scope and the mission of their funding programs. A new platform where funders can search for projects may therefore result in a higher percentage of successful applications. We understand that publishing non-funded applications may lead to some unease among researchers. The academic enterprise is to a substantial part built on competition between researchers and some researchers may consequently not appreciate the publication of their rejected applications. Turning our proposal into a success hence requires a system in which due credit is given to initiators of project ideas. This aligns with calls for more holistic approaches to research evaluation [13,14].

In addition, we argue that, from a normative perspective, the fear of being ‘scooped' is not a valid reason not to engage in sharing funding applications. Based on the combination of the Mertonian norms of *communism* and *universalism*—i.e. any efforts made by science should come to the benefit of the community and only the content of knowledge claims matters, not the one who made them [15]—one should ideally be indifferent as to who is to conduct research along the lines of a grant application, either the original author or someone else. Again, openly sharing applications in fact increases the likelihood of research ideas being put into practice. Despite this advantage for knowledge production, we acknowledge that some applicants may nevertheless perceive this as unfair. However, we argue that using others' research ideas does not constitute ‘stealing ideas' but rather ‘using ideas’ or ‘building on ideas', provided that proper acknowledgements are made. This, we argue, is an inherent part of the research process and of research collaboration. To allow for easy referencing to project proposals, one could consider a system of assigning them a Digital Object Identifier (DOI), similar to what is already common practice for many other forms of scholarly output.

A second set of commonly voiced objections are those addressing the open sharing of reviewer and decision committee member identities, review reports, and argumentations leading to funding decisions, which could lead reviewers and committee members to being less critical out of fear of repercussions. Especially, junior reviewers may worry about the consequences of being critical about grant applications by influential senior colleagues [7]. While these concerns are legitimate, several studies have now addressed them in relation to research funding practices, journal publication or data sharing practices (e.g. [16,17]). These accounts argue that transparency about the assessment of the relevance and quality of applications, including the names of reviewers and decision committee members, could lead to more balanced and more constructive feedback. Even though the evidence for such effects is still inconclusive, the existing evidence does not indicate any sign of less critical evaluations [18]. In addition, experiences with open peer review processes, especially of journal manuscripts, have hitherto been generally positive [19]. If considered too radical, rather than mandating the open sharing of reviewer identities, such decisions could also be left optional, with possibilities for reviewers to opt out, although this would create the risk of self-selection.

## 4. Moving towards open applications

Our plea for more openness in research funding processes is not new. In 2015, Mietchen [20] called for lifting the curtains on the research funding system. Providing several viable routes and discussing some of their strengths and limitations, he already argues for publishing applications as soon as they are submitted, for providing full transparency on evaluation processes and for disclosing evaluators' identities. His plea was inspired by a survey of transparency in grant funding [21] that demonstrated a poor state of affairs at the time of investigation. Only a small minority of a sample of 27 leading global research funders published information regarding reviewer identities, and virtually no funder shared information regarding submitted applications. In fact, one third of the surveyed funders did not even publish the abstracts of successful applications [21].

Since these publications, some progress has been made, but given the time span of nearly a decade, one could easily argue that it has been minimal. Our informal survey of the websites of all funders not publishing abstracts of successful applications by the time of data collection by Gurwitz *et al*. [21] (i.e. nine out of the 27 surveyed funders) indicates that still most of them do not publish such information (six out of nine), while others currently sometimes publish it but only for a fraction of their funding programs. Likewise, the attention for transparency among research funders seems limited within the academic literature. Searching Scopus and Web of Science for (combinations of) the terms ‘open funding’, ‘open applications’, ‘open grants’, ‘open proposals’, ‘open research funding' or ‘open science' AND ‘research funding' only yields a handful of hits, all of which are referenced in this commentary.

However, some funders are taking up the transparency gauntlet and are currently experimenting with some elements of open applications. For example, in its Open Science Fund, the NWO recently asked applicants for consent to publish their proposal. In the first round of the program, NWO received consent to publish 63 applications (or parts thereof) out of the total of 167 that were evaluated [12]. A similar but slightly different initiative was recently established by the British biomedical funder Wellcome. In its Open Research Fund program, it publishes project applications and decision summaries of eligible applications [22].

We understand that funders might be reluctant to change their procedures without clear evidence of the benefits or solid understanding of the implications. Hence, we encourage them to pilot some of the proposed changes described above and evaluate the findings. Needless to say, all such experimentation should be done in the utmost transparent way to allow different organizations to optimally learn from each others' efforts. Such experimentation could study different degrees of openness, exploring in more detail their practical implications, pros and cons of different options of timings, degrees of release of reports, inclusion of author or reviewer identities, or other aspects outlined above. These experiments could evaluate stakeholder experiences, gain insights into potential unintended consequences, and assess levels of uptake and reuse of openly shared material. In addition, it could map collaborative networks and evaluate the extent to which open applications indeed lead to increased or more diverse collaboration.

## 5. Conclusion

In summary, we believe that research funders' central position in the research ecosystem provides them with unique opportunities to optimize knowledge production. The responsibility for the relevance, quality, transparency and trustworthiness of research rests on the shoulders of researchers, research institutes, scholarly journals and research funders alike, with none of them having absolute power [23]. Especially, research funders have the opportunity to set research agendas and to steer norms and practices towards desirable transparent futures. While some front-runners have already acted upon our suggestions for more transparency, the extent to which transparency initiatives permeate the arena of research funders is still minimal and limited to local initiatives and individual funding schemes only. We herald these front-runners and now call on funders more widely to take the next step, in the form of providing transparency about the submitted grant applications and funders' own processes. This could incentivize increased collaboration in the global research system, provide opportunities to more effectively develop and execute relevant research proposals, and allow for more scrutiny of the process of research funding itself. We believe that this would lead to better research and boost both societal trust in research and trust among researchers.

## Data Availability

This article has no additional data.
